# Co-Administration of Roxadustat and Zinc Stabilizes Both Serum Copper and Zinc Concentrations in Patients Undergoing Hemodialysis

**DOI:** 10.3390/nu15234887

**Published:** 2023-11-23

**Authors:** Akira Takahashi

**Affiliations:** Dialysis Center, Tesseikai Neurosurgical Hospital, 28-1 Nakanohonmachi, Shijonawate 575-8511, Japan; kinnereth@gaia.eonet.ne.jp; Tel.: +81-72-877-6639; Fax: +81-72-877-6692

**Keywords:** zinc, hypozincemia, renal anemia, hypoxia-inducible factor-prolyl hydroxylase inhibitor, copper, hypocupremia

## Abstract

Patients undergoing hemodialysis often require zinc supplementation owing to hypozincemia, which may reduce serum copper concentrations. However, hypoxia-inducible factor–prolyl hydroxylase inhibitors (HIF-PHIs), which are used to treat renal anemia, have been reported to increase serum copper. Therefore, this study investigates the effectiveness of a combination of HIF-PHIs and zinc for the stabilization of serum copper and zinc concentrations during zinc supplementation for patients undergoing hemodialysis with renal anemia and hypozincemia. The serum zinc and copper concentrations were retrospectively compared over an 8-month period in 20 patients being administered roxadustat (an HIF-PHI) and 20 controls. The changes in concentrations were tracked in participants taking roxadustat who initiated or increased zinc supplementation. The serum zinc concentrations of the participants were significantly higher (*p* < 0.001) during zinc supplementation, regardless of roxadustat administration. Post-roxadustat, the serum copper concentrations were significantly higher than those pre-roxadustat or in non-roxadustat-treated participants, irrespective of zinc supplementation (*p* < 0.005). Even post-roxadustat, the serum copper concentrations were significantly lower, with no increase during zinc supplementation (*p* < 0.040). When zinc supplementation was initiated or increased in participants taking roxadustat, copper and zinc concentrations were normalized. Thus, combining zinc supplementation with roxadustat prevents both an excessive increase in serum copper and a decrease in serum zinc.

## 1. Introduction

Hypoxia-inducible factor–prolyl hydroxylase inhibitors (HIF-PHIs) activate the HIF oxygen-sensing pathway and promote erythropoiesis through an increase in endogenous erythropoietin production. Although HIF-PHIs are now becoming available for the treatment of renal anemia, side effects have been reported, and recommendations for their appropriate use have been issued [1]. Recently, there has been concern regarding the development of high serum copper concentrations following the commencement of HIF-PHI use [2]. Gastrointestinal discomfort, diarrhea, and vomiting have been reported as gastrointestinal side effects of HIF-PHIs [3], but the same symptoms also develop in patients with copper excess [4]. Therefore, an HIF-PHI-induced high serum copper concentration may be the cause of these gastrointestinal symptoms.

Most patients undergoing hemodialysis require zinc supplementation because of hypozincemia [5], which is associated with various diseases. In addition, meta-analyses have shown that zinc supplementation improves the nutritional status of patients undergoing hemodialysis [6]. However, there is concern that long-term zinc supplementation may reduce serum copper concentrations because of the induction of metallothionein [7]. Therefore, it is conceivable that the use of a combination of an HIF-PHI and zinc supplementation could prevent excessive increases in serum copper concentration when using HIF-PHIs and also prevent the decrease in serum copper concentration caused by zinc supplementation. In addition, regarding vascular calcification, which is a concern when using HIF-PHIs, it has been reported that the calcification caused by HIF-PHI is suppressed by zinc in vitro [8], and it is thought that there would be an advantage in supplementing zinc together when using an HIF-PHI.

Furthermore, regarding cognitive impairment, there have been previous reports that hypocupremia causes Alzheimer’s disease [9], and people with high copper levels have been shown to have a lower risk of developing Alzheimer’s disease [10]. The use of HIF-PHIs may also increase serum copper concentrations in patients with hypocupremia, which may also help prevent Alzheimer’s disease. However, the excessive intake of copper can have detrimental effects on the brain [11], and some previous studies have shown that patients with Alzheimer’s disease have significantly higher serum copper concentrations than healthy individuals. Therefore, it is necessary that the serum copper concentrations of patients are neither too high nor too low [12].

Appropriate zinc supplementation can prevent excessive increases in serum copper concentration [13,14]. However, there have been reports that zinc suppresses amyloid-β aggregation [15], which causes Alzheimer’s disease, and also that zinc promotes amyloid-β aggregation [16,17]. Therefore, the supplementation of zinc as a method of adjusting the serum copper concentration should be carried out carefully, not only to prevent hypocupremia but also to prevent hyperzincemia.

The aim of the present study was to determine whether simultaneous zinc supplementation and administration of an HIF-PHI would normalize the serum copper and zinc concentrations during zinc supplementation of patients undergoing hemodialysis who have renal anemia and hypozincemia.

## 2. Methods

### 2.1. Participants

A retrospective study was performed in outpatients who were undergoing maintenance hemodialysis in the morning. Those undergoing hemodialysis in the afternoon were excluded because the circadian rhythm of serum zinc concentration involves a peak in the morning and a decrease of approximately 20% in the afternoon [18]. Patients who avoided the use of HIF-PHIs or used them with caution for the treatment of renal anemia (those with malignant tumors, retinopathy, liver dysfunction, or polycystic kidney disease) were excluded, as were patients with hemorrhagic lesions. All the participants received standard dietary advice for patients undergoing hemodialysis, but no further advice was provided regarding their diet before the commencement of hemodialysis.

Twenty patients who had taken an HIF-PHI (roxadustat) for 2 years since May 2020 were included in the study, and 20 who had not taken an HIF-PHI during the same period were used as controls. The baseline characteristics of the participants after applying the exclusion criteria are shown in Table 1.

### 2.2. Observation Period

HIF-PHI-treated participants were retrospectively monitored for 8 months before HIF-PHI administration (pre) and for 8 months after its commencement (post), and comparisons were made with controls who were not taking an HIF-PHI during the same period (16 months, during which data were collected on six occasions).

### 2.3. Measurements

**Figure 1:**
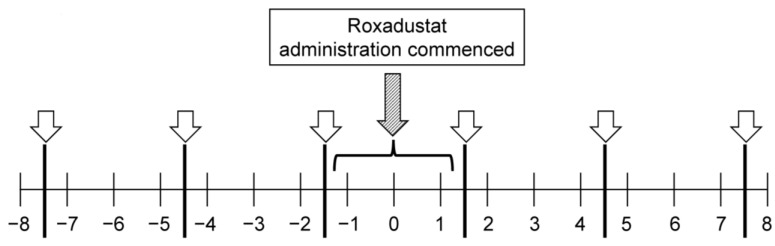
Observation period and timing of the measurement of the serum copper and zinc concentrations. The observation period comprised 8 months both before and after the start of HIF-PHI administration. Because the serum copper and zinc concentrations were routinely measured every 3 months (white arrow), these data were obtained three times both before and after starting HIF-PHI administration. Roxadustat administration commenced during the 3-month period indicated by the diagonal arrow.

The serum zinc and copper concentrations of the participants were measured every 3 months as part of routine clinical practice; therefore, these concentrations were measured three times during each of the 8-month periods (Figure 1). Serum zinc concentration was measured using a direct colorimetric assay, based on the nitro-PAPS method, using a JCA-BM6050 BioMajesty (JEOL Ltd., Tokyo, Japan) and ESPA ZnII (Nipro Co., Ltd., Osaka, Japan) reagent. Serum copper concentration was measured using a direct colorimetric assay, based on the 3,5-DiBr-PAESA method, and the JCA-BM6050 BioMajesty and Quick Auto Neo Cu (Shino-Test, Tokyo, Japan) were used as the reagents. The normal ranges for the serum copper and zinc concentrations were 11.2–20.8 µmol/L (71–132 µg/dL) and 12.2–19.9 µmol/L (80–130 µg/dL), respectively [19,20].

### 2.4. Data Collection

The dates of the start of drug administration and blood sample collection differed; therefore, the serum zinc and copper concentrations that were measured during the administration of roxadustat and zinc supplementation for more than a week were regarded as being representative of the status of each mineral during the administration of roxadustat and zinc supplementation.

To compare the effects of zinc, participants were selected who had both periods of zinc supplementation and periods of no zinc supplementation.

Roxadustat-treated participants who had both a period of zinc supplementation and a period without it and had their serum copper and zinc concentrations measured three times during the 8-month observation period were identified, and their data were regarded as reflecting the post-roxadustat status. In addition, these roxadustat-treated participants were similarly sampled during the 8 months prior to roxadustat administration, and the data obtained were regarded as reflecting pre-roxadustat status. Finally, participants who did not take roxadustat during the same period (16 months) but who experienced periods both with and without zinc supplementation were identified, and their data were regarded as reflecting no-roxadustat status.

**Figure 2:**
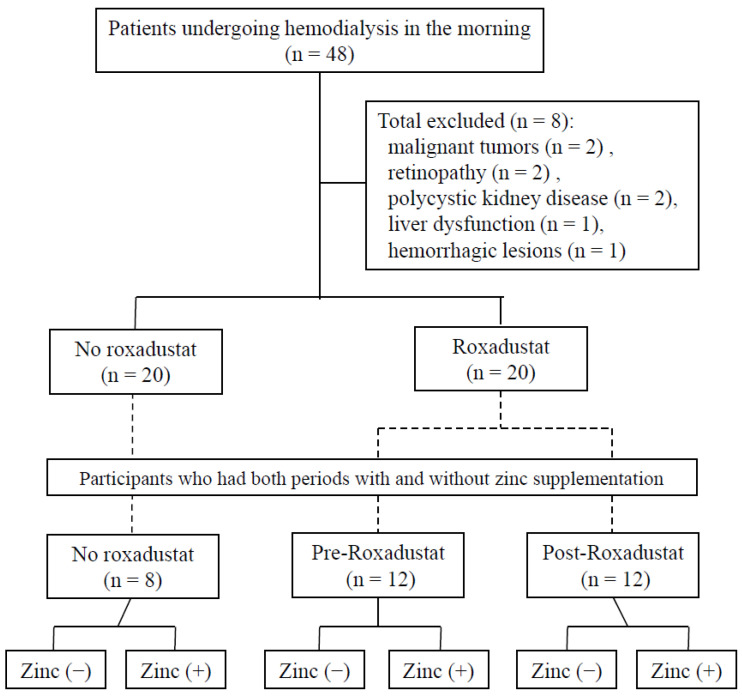
Study design: pre-roxadustat, data obtained during the 8 months prior to roxadustat administration; post-roxadustat, data obtained during the 8 months after roxadustat administration commenced; no roxadustat, data obtained during the same 16-month period as the post-roxadustat period in participants who had not administered roxadustat; zinc (+), data obtained during the period of zinc supplementation; zinc (−), data obtained during the period of no zinc supplementation.

Figure 2 shows the number of participants for whom data were obtained in each group. The number of data points obtained for each participant who was or was not supplementing zinc during each of the study periods differed.

### 2.5. Statistical Analyses

Data are presented as the mean ± standard deviation for continuous data and percentages and counts for categorical data. Microsoft 365 Excel (Microsoft Corporation, Redmond, WA, USA) was used for data analysis. The baseline characteristics of the two groups were compared using the two-sample Student’s *t* test, assuming unequal variances. Effect sizes were compared using Cohen’s *d*. The three groups that yielded differing numbers of data were compared using one-way ANOVA. Categorical data were compared using the chi-square test. Relationships between continuous data were evaluated using Pearson’s product-moment correlation coefficient, calculated using the CORREL function, with *p* values being obtained using the TDIST function. Statistical significance was set at *p* < 0.05.

### 2.6. Treatments

Roxadustat (Astellas Pharma Inc., Tokyo, Japan; 20-, 50-, and 100-mg tablets) was the HIF-PHI used. After switching from an erythropoiesis-stimulating agent (ESA) to roxadustat for the treatment of renal anemia, the doses of iron and roxadustat administered were adjusted in accordance with the recommendations of the Asia Pacific Society of Nephrology (APSN) regarding the appropriate use of HIF-PHIs [1] and the Guidelines for Renal Anemia in Chronic Kidney Disease [21]. Because thromboembolism has been reported to be a complication of HIF-PHI administration that is associated with iron deficiency [22,23], iron was supplemented when the serum ferritin concentration of a participant was <100 µg/L or their transferrin saturation was <20% during roxadustat administration.

Since 2018, serum zinc and copper concentrations have been measured at the hospital every 3 months as part of the routine blood testing of patients undergoing hemodialysis, and based on that experience, the following zinc supplementation protocol was adopted. Zinc supplementation was initiated with zinc acetate hydrate (molecular formula: C_4_H_6_O_4_Zn.2H_2_O) 50 mg/day (zinc content 50 mg) in participants with frank (serum zinc concentration < 9.2 µmol/L) zinc deficiency, polaprezinc (molecular formula: C_9_H_12_N_4_O_3_Zn) 150 mg/day (zinc content 34 mg) was initiated in participants with marginal (serum zinc concentration 9.2–12.2 µmol/L) zinc deficiency, and the target serum zinc concentration was set as 12.2–18.4 µmol/L [13]. When the serum zinc concentrations of the patients reached ≥15.3 µmol/L, the level of zinc supplementation was reduced to 25 mg, and it was discontinued when the serum concentrations were ≥18.4 µmol/L. The serum zinc and copper concentrations of the patients were measured before the commencement of zinc supplementation, as well as every 3 months afterwards, and the values obtained were used to adjust the level of zinc supplementation.

Sevelamer hydrochloride, a therapy for hyperphosphatemia, is highly adsorptive of copper and therefore affects the serum copper concentrations of patients undergoing dialysis [24]. Specifically, it has been reported to adsorb copper and zinc ions at pH 6.8, with adsorption ratios of 99% and 38%, respectively [25]. Therefore, the use of sevelamer hydrochloride was also investigated.

## 3. Results

### 3.1. Characteristics of the Participants

During the observation period, of the 20 participants undergoing dialysis (9 men and 11 women) in the morning who were taking roxadustat, data for 12 who had experienced both periods with and without zinc supplementation were collected. During the period before roxadustat administration, 20 sets of data were collected from these 12 participants, comprising their serum copper and zinc concentrations, their dose of roxadustat, and their level of zinc supplementation during the periods of no zinc supplementation, and 21 sets of data were obtained during periods of zinc supplementation. During the period following the start of roxadustat administration in these 12 participants, 10 sets of data were obtained for periods of no zinc supplementation, and 18 sets were obtained for periods of zinc supplementation.

Of the 20 participants (11 men and 9 women) who had not taken roxadustat during the same period, 8 who had periods both with and without zinc supplementation were used as controls. During this period, 11 sets of data were obtained for periods of no zinc supplementation, and 13 sets were obtained for periods of zinc supplementation for these participants.

Table 2 shows the baseline characteristics of these participants.

### 3.2. Serum Zinc and Copper Concentrations of Participants Who Were or Were Not Administering Roxadustat

The serum zinc concentrations of the participants were significantly higher (*p* < 0.001) during zinc supplementation, regardless of whether roxadustat was being administered or not.

The serum copper concentrations post-roxadustat were significantly higher than those pre-roxadustat and those in participants who had not administered roxadustat, irrespective of zinc supplementation (*p* < 0.005) (Table 3).

**Figure 3:**
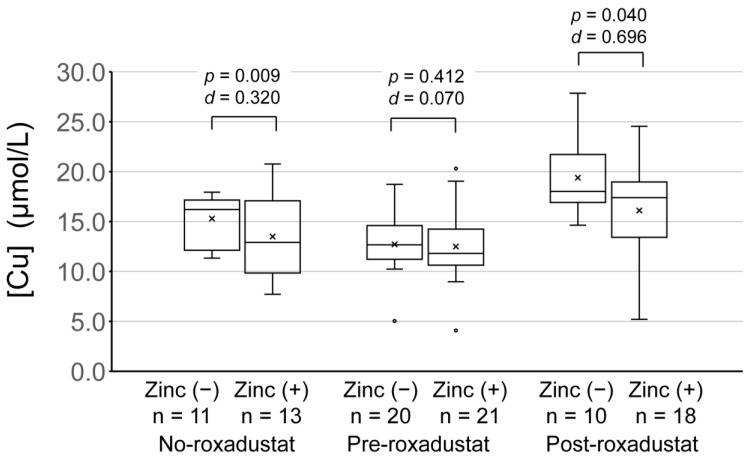
[Cu] in the period of zinc supplementation and the period of no supplementation in participants who did not administer roxadustat and pre- and post-roxadustat in those who did. The baseline characteristics of the two groups were compared using the two-sample Student’s *t* test, assuming unequal variances. Effect size was compared using Cohen’s *d*. [Cu], serum copper concentration; pre-roxadustat, data obtained during the 8 months prior to roxadustat administration; post-roxadustat, data obtained during the 8 months after roxadustat administration commenced; no roxadustat, data obtained during the same 8 months as the post-roxadustat period in participants who did not administer roxadustat; zinc (+), data obtained during the period of zinc supplementation; zinc (−), data obtained during the period of no zinc supplementation.

These data were then analyzed according to whether or not zinc was being supplemented (Figure 3). The serum copper concentrations of participants pre-roxadustat were not lower when zinc was being supplemented (*p* = 0.412). However, there were significantly lower serum copper concentrations during zinc supplementation than during periods of no zinc supplementation in the participants who had not been administered roxadustat (*p* = 0.009) and in those who were post-roxadustat (*p* = 0.040). The effect size for the lowering of serum copper concentration alongside zinc administration, indicated by Cohen’s *d*, was larger for the post-roxadustat group (*d* = 0.696) than for the no-roxadustat group (*d* = 0.320).

### 3.3. Relationship between Roxadustat Dose and Serum Copper Concentration

The relationship between the roxadustat dose and serum copper concentrations of the participants was evaluated and no correlation between these two variables was found (*r* = 0.089, *p* = 0.590).

The highest serum copper concentration measured was 29.0 µmol/L, and serum concentrations above the upper limit of the normal range of 20.8 µmol/L were obtained for six participants. However, the serum copper concentrations of these participants returned to normal after increasing their level of zinc supplementation, and they did not show symptoms of copper excess, such as gastrointestinal symptoms.

### 3.4. Assessment of the Level of Zinc Supplementation

Increasing the level of zinc supplementation increases the metallothionein concentrations of patients and reduces those of copper [26]; therefore, the level of zinc supplementation being used was assessed. The zinc content of the zinc acetate hydrate administered was 25 or 50 mg, and that of polaprezinc was 17 mg/tablet; therefore, the participants were administered 34 mg/day. There was a significant difference in the level of zinc supplementation among the no roxadustat, pre-roxadustat, and post-roxadustat groups (*p* = 0.026). There was no difference in zinc supplementation between the pre-roxadustat and post-roxadustat groups. However, participants in the post-roxadustat group were supplemented with significantly more zinc than those in the non-roxadustat group (Table 3).

### 3.5. Normalization of High Serum Copper and Low Serum Zinc Concentrations Following the Initiation of, or an Increase in, the Level of Zinc Supplementation

**Figure 4:**
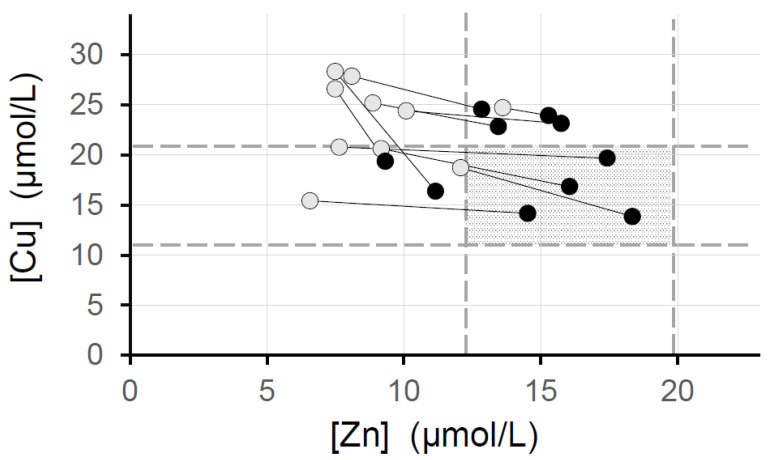
Amelioration of hypocupremia and hypozincemia by the use of a combination of roxadustat and zinc supplementation. ○, before the administration of roxadustat; ●, following the commencement of roxadustat administration. [Cu], serum copper concentration (normal range 11.2–20.8 µmol/L); [Zn], serum zinc concentration (normal range 12.2–19.9 µmol/L).

Of the participants who were administered roxadustat, 11 either initiated zinc supplementation or increased their level of supplementation during the observation period. The changes that occurred in their serum copper and zinc concentrations are shown in Figure 4. After the initiation of, or an increase in, the level of zinc supplementation, their serum copper and zinc concentrations were within or close to the respective reference ranges in all 11 cases. Their serum zinc concentrations increased from 9.1 ± 2.1 to 13.8 ± 3.0 µmol/L, and their serum copper concentrations decreased from 23.5 ± 4.2 to 19.8 ± 4.1 µmol/L (mean ± standard deviation).

## 4. Discussion

In the present study, it was shown that the use of HIF-PHIs increases the serum copper concentrations of patients, as reported by Nakamura et al. [2]. However, zinc supplementation can reduce serum copper concentrations through the induction of metallothioneins [7]. Therefore, in patients taking an HIF-PHI, roxadustat, it was expected that those who were supplementing zinc would have lower serum copper concentrations than those who were not. In patients taking an HIF-PHI, serum copper concentrations were shown to be significantly lower in those who were supplementing zinc. No correlation between the dose of roxadustat administered and the serum copper concentration of patients was identified. This indicates that even small doses of roxadustat can increase serum copper concentrations.

Since 2018, the serum zinc and copper concentrations of patients undergoing hemodialysis have been routinely measured every 3 months at the hospital, and on the basis of that experience, the zinc supplementation protocol described in the Treatments section was adopted. The serum zinc and copper concentrations of each participant were measured every 3 months during the present study, and the level of zinc supplementation was adjusted appropriately. Therefore, it was possible to increase the amount of zinc supplementation in patients who were taking roxadustat after confirming that their serum copper concentrations did not decrease alongside the use of roxadustat but instead increased. If the serum copper concentration of a participant was high, it returned to within or close to the normal range following the initiation or an increase in the level of zinc supplementation (Figure 4). Therefore, when using HIF-PHIs, an excessive increase in serum copper concentration can be prevented by anticipating the rise in serum copper concentration and providing an appropriate dose of zinc in advance.

The serum zinc concentrations of patients with renal failure tend to be low because of inadequate intake, malabsorption, or excessive loss of zinc. Therefore, most patients who undergo hemodialysis have frank or marginal zinc deficiency [27]. If a patient’s serum zinc concentration is low, zinc administration may improve this and ameliorate the symptoms of the underlying disease. Therefore, even if symptoms of zinc deficiency are not being reported, supplementation is recommended if hypozincemia is identified [20]. Because zinc supplementation reduces the serum copper concentrations of patients, owing to its effect on metallothionein [26], the level of zinc supplementation should be adjusted appropriately. However, although the need for zinc supplementation is recognized, it is difficult to provide sufficient zinc over the long term because of the high risk of hypocupremia. Encouragingly, in the present study, it was shown that concomitant zinc supplementation when using an HIF-PHI not only prevents a decrease in serum copper concentration but also an excessive increase in serum copper.

**Figure 5:**
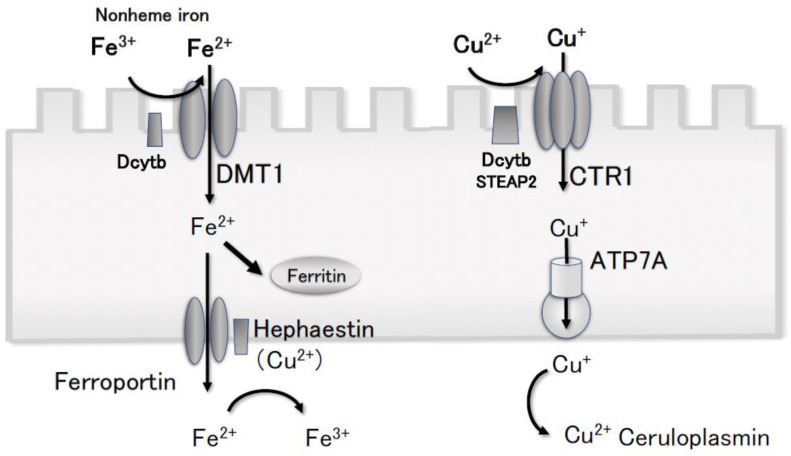
The increase in serum copper concentration in HIF-PHI users is caused by an increase in the expression of metal transporters. Hypoxia-inducible factor-α increases the expression of DMT1 and Dcytb [27], which are involved in the absorption of divalent cations, such as copper and zinc. Hypoxia-inducible factor-α increases the expression of the copper transporters CTR1 [29,30] and ATP7A [31], and copper is reduced from the divalent to the monovalent form in the duodenum, where it is absorbed. This figure has been modified and reprinted with permission [13]. Dcytb, duodenal cytochrome b; DMT1, divalent metal transporter 1; STEAP2, six-transmembrane epithelial antigen of prostate-2; ATP7A, ATPase 7A.

The explanation for the increase in serum copper concentration in HIF-PHI users is an HIF-2α-induced increase in the expression of divalent metal transporter 1 (DMT1) and duodenal cytochrome b [28], which are involved in the absorption of divalent cations such as iron, copper, and zinc. HIF-2α increases the expression of ferroportin [29]. In addition, HIF-2α increases the expression of the copper transporters CTR1 [30,31] and ATPase 7A [32], which are involved in the absorption of monovalent copper in the duodenum (Figure 5).

Untreated hypercupremia in patients undergoing dialysis can cause oxidative stress, leading to dyslipidemia, the exacerbation of inflammation [24], cardiovascular disease [33], and a higher risk of death [34]. Therefore, it may be important to supplement zinc intake to reduce the serum copper concentrations of patients. Conversely, because copper plays an important role in many metabolic processes, including cellular respiration, iron oxidation, and hemoglobin synthesis, acquired copper deficiency can lead to abnormalities such as myelodysplasia and pancytopenia [35]. However, there is no effective treatment for hypocupremia other than copper injections and oral treatment [36].

Zinc is a cofactor in more than 300 enzymes, is part of the structure of numerous zinc finger proteins, and is involved in signaling in various tissues. Therefore, hypozincemia can present in a number of ways [37]. Zinc is used clinically for its anti-inflammatory and antioxidant effects at various sites [38] and is essential for the transcriptional function of nuclear factor erythroid 2-related factor 2 [39]. In addition, many studies have shown a relationship between zinc and diabetes, and zinc supplementation improves the HbA1c values and fasting blood glucose concentrations of patients [40].

Regarding cognitive impairment, no definitive results have yet been obtained regarding the reduction of dementia risk through diet and nutrition [41,42]. Some previous studies have shown that high copper intake is associated with a higher risk of dementia [43], while others have identified no such association [44]. It has been reported that a diet high in copper, especially when combined with a high saturated fat content, may increase the risk of cognitive impairment [45]. These findings imply that the relationship between copper status and the risk of developing dementia is complex.

At the International Conference on Nutrition and the Brain, Washington, DC, 19–20 July 2013, speakers were asked to comment on potential guidelines for the prevention of Alzheimer’s disease. They commented that excessive copper (or iron) intake may contribute to Alzheimer’s disease and suggested the use of multivitamin/mineral supplements that do not contain these minerals [46]. Therefore, it is very important to maintain a balanced copper intake, and special attention is required in the prevention and progression of dementia.

With respect to renal anemia, not only zinc but also copper is necessary for the function of many regulators of hematopoiesis, such as growth hormone, insulin-like growth factor 1, GATA-binding protein 1, vitamin D receptor, hephaestin, and copper–zinc superoxide dismutase [13]. The required dose of ESA can be reduced by instituting an appropriate level of zinc supplementation [47].

**Figure 6:**
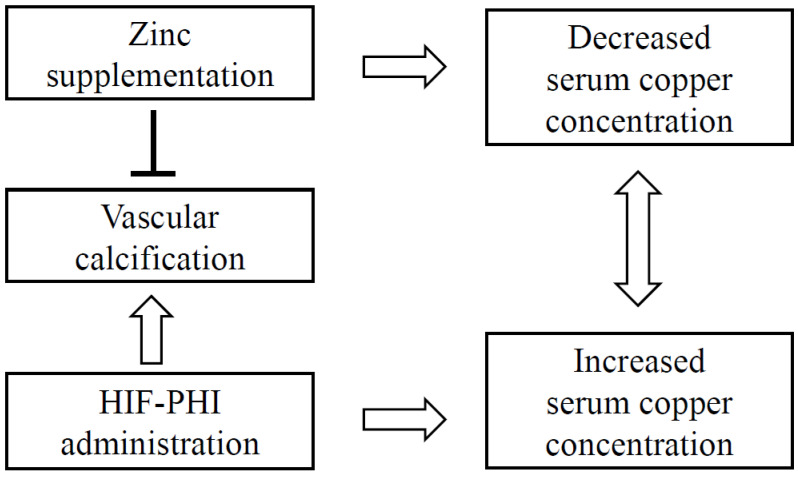
Use of a combination of an HIF-PHI and zinc supplementation. Hypozincemia is common in patients undergoing hemodialysis [5], and zinc supplementation is recommended when hypozincemia is identified [6]. Hypoxia-inducible factor 1-α strongly promotes phosphate-induced vascular smooth muscle cell calcification [48]. Therefore, HIF-PHIs may exacerbate vascular calcification. However, this calcification may be reduced by adequate zinc supplementation [49,50]. If zinc supplementation is required when an HIF-PHI is administered, concern regarding an increase in [Cu] owing to HIF-PHI administration and a decrease in [Cu] owing to zinc supplementation can be alleviated. The combination of HIF-PHI and zinc supplementation is safe and effective. HIF-PHI, hypoxia-inducible factor-prolyl hydroxylase inhibitor; [Cu], serum copper concentrations.

Zinc contributes to bone mineralization by inhibiting bone resorption and promoting bone formation [48], but it also inhibits vascular calcification. HIF-PHIs exacerbate vascular calcification because HIF-1α promotes phosphate-induced vascular smooth muscle cell calcification [49]. Zinc also inhibits the calcification of vascular smooth muscle cells that is induced by HIF-1α [8]. Zinc acts via G-protein-coupled receptor 39 to induce the expression of tumor necrosis factor-alpha-induced protein 3 and inhibits nuclear factor κB, thereby reducing phosphate-induced vascular calcification [50]. Therefore, zinc would be expected to prevent calcification when administered to patients whose renal anemia is being treated using an HIF-PHI. In the present study, it was shown that the increase in serum copper concentration caused by the administration of an HIF-PHI can be used to balance the serum zinc and copper concentrations of patients during zinc supplementation. In other words, it was shown that the increase in serum copper concentration owing to HIF-PHI administration is offset by the decrease in copper concentration caused by zinc supplementation (Figure 6). In addition, the use of zinc supplementation alongside the administration of an HIF-PHI prevented the reduction in serum copper concentration caused by zinc supplementation and was found to be safe.

During zinc supplementation, in anticipation of the associated decrease in serum copper concentration, dietary guidance regarding how patients could increase their serum copper concentrations should be provided. This would include a recommendation to consume copper-rich nuts and hemocyanin-containing seafood, such as squid, octopus, shrimp, clams, and crabs. However, when patients are taking an HIF-PHI, they should reduce their intake of copper, and the early introduction of dietary adjustment and zinc supplementation are important in preventing a deleterious increase in serum copper concentration.

The present study had some limitations. First, the level of zinc supplementation of the participants was changed according to the zinc supplementation protocol described in the Methods section on the basis of their serum copper and zinc concentrations, measured every 3 months. The dose of HIF-PHI was then adjusted according to their anemia status twice a month. Therefore, data were retrospectively extracted from medical records under circumstances in which factors influencing the serum copper concentration were constantly changing. In addition, the final analysis was performed on patients who had experienced periods of both zinc supplementation and no zinc supplementation, which reduced the number of data points obtained. In particular, the number of data points obtained before the use of an HIF-PHI commenced was as small as 10 for participants using an HIF-PHI. However, if the observation period had been extended beyond 8 months (three data points), in order to increase the amount of data collected, all the patients using an HIF-PHI might have been treated by zinc supplementation alone. Therefore, it was not possible to extend the observation period.

Although the study was conducted under conditions during which the factors that affect serum copper concentration, which are cited as a limitation, were constantly changing, one of the strengths of this study was that a zinc supplementation protocol had been introduced in 2018. Because the level of zinc supplementation was already being adjusted according to this protocol, even when changes in serum copper concentration secondary to the use of an HIF-PHI were superimposed upon the changes in the serum copper and zinc concentrations that occur during zinc supplementation, it may be considered that the investigation of the effects of HIF-PHI was conducted in a stable environment.

## 5. Conclusions

The use of a combination of HIF-PHI administration and zinc supplementation not only prevents the excessive increase in serum copper concentration during HIF-PHI administration but also enables safe zinc supplementation without a reduction in serum copper concentration in patients undergoing hemodialysis who are at a high risk of zinc deficiency.

## Figures and Tables

**Table 1:** Baseline characteristics of the participants after the application of the exclusion criteria.

	No Roxadustat	Roxadustat	*p* Value
Number of participants	20	20	
Male sex, n (%)	8 (40.0)	12 (60.0)	0.206
Age, mean ± SD (years)	77.1 ± 12.3	73.0 ± 11.4	0.274
Duration of dialysis, mean ± SD (years)	7.0± 5.2	6.9 ± 4.7	0.953
Etiology, n (%)			0.725
Diabetic nephropathy	9 (45.0)	7 (35.0)	
Nephrosclerosis	7 (35.0)	8 (40.0)	
Chronic glomerulonephritis	2 (10.0)	4 (20.0)	
Other	2 (10.0)	1 (5.0)	

No roxadustat, participants who did not administer roxadustat; roxadustat, participants who administered roxadustat at any time during the study.

**Table 2:** Baseline characteristics of the participants who had both periods with and without zinc supplementation.

	No Roxadustat	Roxadustat	*p* Value
Number of participants	8	12	
Male sex, n (%)	5 (62.5)	6 (50.0)	0.582
Age, mean ± SD (years)	81.8 ± 6.6	76.0 ± 6.1	0.061
Duration of dialysis, mean ± SD (years)	7.2 ± 5.0	7.3± 5.6	0.970
Etiology, n (%)			0.222
Diabetic nephropathy	5 (62.5)	4 (33.3)	
Nephrosclerosis	1 (12.5)	6 (50.0)	
Chronic glomerulonephritis	1 (12.5)	2 (16.7)	
Uncertain	1 (12.5)	0 (0.00)	

No roxadustat, participants who did not administer roxadustat; roxadustat, participants who administered roxadustat at any time during the study.

**Table 3:** [Cu] and [Zn] in participants who were or were not taking roxadustat and were or were not supplementing zinc.

		No Roxadustat	Pre-Roxadustat	Post-Roxadustat	ANOVA *p*-Value	Roxadustat Dose (mg)
[Zn] (µmol/L)	Zinc (−)	9.6 ± 1.2	8.6 ± 1.6	7.0 ± 1.4	<0.001	59.0 ± 26.0
	Zinc (+)	13.1 ± 4.1	11.1 ± 2.1	10.6 ± 3.17	0.068	59.4 ± 25.5
	*p*-value	<0.001	<0.001	<0.001		0.483
[Cu] (µmol/L)	Zinc (−)	15.3 ± 2.47	12.7 ± 2.8	19.4 ± 4.1	<0.005	
	Zinc (+)	12.4 ± 3.1	12.5 ± 3.5	16.1 ± 5.3	<0.005	
	*p*-value	0.009	0.412	0.040		
	Cohen’s *d*	0.320	0.070	0.696		
Zinc dose (mg)		28.5 ± 4.6	35.1 ± 10.4	36.2 ± 6.7 *	0.026	

* *p* < 0.001 vs. the No Roxadustat group. [Cu], serum copper concentration (normal range 11.2–20.8 µmol/L); [Zn], serum zinc concentration (normal range 12.2–19.9 µmol/L); No roxadustat, participants who did not take roxadustat; pre-roxadustat, participants before roxadustat administration; post-roxadustat, participants after roxadustat administration commenced; zinc (+), data obtained during the period with zinc supplementation; zinc (−), data obtained during the period without zinc supplementation.

## Data Availability

The data that support the findings of this study will be made available from the corresponding author upon reasonable request.

## Abbreviations

HIF-PHI: hypoxia-inducible factor–prolyl hydroxylase inhibitor; ESA, erythropoiesis-stimulating agent; DMT1, divalent metal transporter 1.

## References

[1] Yap D.Y.H., McMahon L.P., Hao C., Hu N., Okada H., Suzuki Y., Kim S.G., Lim S.K., Vareesangthip K., Hung C. (2021). APSN HIF-PHI Recommendation Committee. Recommendations by the Asian Pacific Society of Nephrology (APSN) on the appropriate use of HIF-PH inhibitors. Nephrology.

[2] Nakamura H., Kurihara S., Anayama M., Makino Y., Nagasaw M. (2022). Four cases of serum copper excess in patients with renal anemia receiving a hypoxia-inducible factor-prolyl hydroxylase inhibitor: A possible safety concern. Case Rep. Nephrol. Dial..

[3] Post-Marketing Surveillance of EVRENZO® Tablets (Roxadustat) in Patients with Renal Anemia. ClinicalTrials.gov Identifier: NCT04408820. [Internet]. NCT04408820.

[4] Knobeloch L., Ziarnik M., Howard J., Theis B., Farmer D., Anderson H., Proctor M. (1994). Gastrointestinal upsets associated with ingestion of copper-contaminated water. Environ. Health Perspect..

[5] Bozalioğlu S., Ozkan Y., Turan M., Simşek B. (2005). Prevalence of zinc deficiency and immune response in short-term hemodialysis. J. Trace Elem. Med. Biol..

[6] Wang L.-J., Wang M.-Q., Hu R., Yang Y., Huang Y.-S., Xian S.-X., Lu L. (2017). Effect of zinc supplementation on maintenance hemodialysis patients: A systematic review and meta-analysis of 15 randomized controlled trials. Biomed. Res. Int..

[7] Fischer P.W., Giroux A., L’Abbé M.R. (1981). The effect of dietary zinc on intestinal copper absorption. Am. J. Clin. Nutr..

[8] Nagy A., Pethő D., Gáll T., Zavaczki E., Nyitrai M., Posta J., Zarjou A., Agarwal A., Balla G., Balla J. (2020). Zinc inhibits HIF-prolyl hydroxylase inhibitor-aggravated VSMC calcification induced by high phosphate. Front. Physiol..

[9] Klevay L.M. (2008). Alzheimer’s disease as copper deficiency. Med. Hypotheses.

[10] Siotto M., Simonelli I., Pasqualetti P., Mariani S., Caprara D., Bucossi S., Ventriglia M., Molinario R., Antenucci M., Rongioletti M. (2016). Association Between Serum Ceruloplasmin Specific Activity and Risk of Alzheimer’s Disease. J. Alzheimer’s Dis..

[11] Royer A., Sharman T. (2023). Copper Toxicity [Updated 27 March 2023]. StatPearls [Internet].

[12] Squitti R., Simonelli I., Ventriglia M., Siotto M., Pasqualetti P., Rembach A., Doecke J., Bush A.I. (2014). Meta-analysis of serum non-ceruloplasmin copper in Alzheimer’s disease. J. Alzheimer’s Dis..

[13] Takahashi A. (2022). Role of zinc and copper in erythropoiesis in patients on hemodialysis. J. Ren. Nutr..

[14] Nishime K., Kondo M., Saito K., Miyawaki H., Nakagawa T. (2020). Zinc Burden Evokes Copper Deficiency in the Hypoalbuminemic Hemodialysis Patients. Nutrients.

[15] Garai K., Sahoo B., Kaushalya S.K., Desai R., Maiti S. (2007). Zinc lowers amyloid-beta toxicity by selectively precipitating aggregation intermediates. Biochemistry.

[16] Miller Y., Ma B., Nussinov R. (2010). Zinc ions promote Alzheimer Abeta aggregation via population shift of polymorphic states. Proc. Natl. Acad. Sci. USA.

[17] Lee M.C., Yu W.C., Shih Y.H., Chen C.Y., Guo Z.H., Huang S.J., Chan J.C.C., Chen Y.R. (2018). Zinc ion rapidly induces toxic, off-pathway amyloid-β oligomers distinct from amyloid-β derived diffusible ligands in Alzheimer’s disease. Sci. Rep..

[18] Kanabrocki E.L., Sothern R.B., Ryan M.D., Kahn S., Augustine G., Johnson C., Foley S., Gathing A., Eastman G., Friedman N. (2008). Circadian characteristics of serum calcium, magnesium and eight trace elements and of their metallo-moieties in urine of healthy middle-aged men. Clin. Ter..

[19] Shibata S., Kitamura M. (1964). Modern diagnostic testing system. Routine Clinical Biochemical Quantitative Methods.

[20] Kodama H., Tanaka M., Naito Y., Katayama K., Moriyama M. (2020). Japan’s practical guidelines for zinc deficiency with a particular focus on taste disorders, inflammatory bowel disease, and liver cirrhosis. Int. J. Mol. Sci..

[21] Yamamoto H., Nishi S., Tomo T., Masakane I., Saito K., Nangaku M., Hattori M., Suzuki T., Morita S., Ashida A. (2017). 2015 Japanese Society for Dialysis Therapy: Guidelines for renal anemia in chronic kidney disease. Ren. Replace. Ther..

[22] Tang X., Zhang Z., Fang M., Han Y., Wang G., Wang S., Xue M., Li Y., Zhang L., Wu J. (2020). Transferrin plays a central role in coagulation balance by interacting with clotting factors. Cell Res..

[23] Tang X., Fang M., Cheng R., Zhang Z., Wang Y., Shen C., Han Y., Lu Q., Du Y., Liu Y. (2020). Iron-Deficiency and Estrogen Are Associated With Ischemic Stroke by Up-Regulating Transferrin to Induce Hypercoagulability. Circ. Res..

[24] Ikee R., Tsunoda M., Sasaki N., Sato N., Hashimoto N. (2013). Clinical factors associated with serum copper levels and potential effect of sevelamer in hemodialysis patients. Int. Urol. Nephrol..

[25] Takagi K., Masuda K., Yamazaki M., Kiyohara C., Itoh S., Wasaki M., Inoue H. (2010). Metal ion and vitamin adsorption profiles of phosphate binder ion-exchange resins. Clin. Nephrol..

[26] Suzuki K.T., Maitani T. (1981). Metal-dependent properties of metallothionein. Replacement in vitro of zinc in zinc-thionein with copper. Biochem. J..

[27] Nakatani S., Mori K., Shoji T., Emoto M. (2021). Association of Zinc Deficiency with Development of CVD Events in Patients with CKD. Nutrients.

[28] Mastrogiannaki M., Matak P., Keith B., Simon M.C., Vaulont S., Peyssonnaux C. (2009). HIF-2alpha, but not HIF-1alpha, promotes iron absorption in mice. J. Clin. Investig..

[29] Taylor M., Qu A., Anderson E.R., Matsubara T., Martin A., Gonzalez F.J., Shah Y.M. (2011). Hypoxia-inducible factor-2α mediates the adaptive increase of intestinal ferroportin during iron deficiency in mice. Gastroenterology.

[30] White C., Kambe T., Fulcher Y.G., Sachdev S.W., Bush A.I., Fritsche K., Lee J., Quinn T.P., Petris M.J. (2009). Copper transport into the secretory pathway is regulated by oxygen in macrophages. J. Cell Sci..

[31] Pourvali K., Matak P., Latunde-Dada G.O., Solomou S., Mastrogiannaki M., Peyssonnaux C., Sharp P.A. (2012). Basal expression of copper transporter 1 in intestinal epithelial cells is regulated by hypoxia-inducible factor 2α. FEBS Lett..

[32] Xie L., Collins J.F. (2011). Transcriptional regulation of the Menkes copper ATPase (Atp7a) gene by hypoxia-inducible factor (HIF2{alpha}) in intestinal epithelial cells. Am. J. Physiol. Cell Physiol..

[33] Ari E., Kaya Y., Demir H., Asicioglu E., Keskin S. (2011). The correlation of serum trace elements and heavy metals with carotid artery atherosclerosis in maintenance hemodialysis patients. Biol. Trace Elem. Res..

[34] Tonelli M., Wiebe N., Bello A., Field C.J., Gill J.S., Hemmelgarn B.R., Holmes D.T., Jindal K., Klarenbach S.W., Manns B.J. (2018). Concentrations of trace elements and clinical outcomes in hemodialysis patients: A prospective cohort study. CJASN.

[35] Tahir N., Ashraf A., Waqar S.H., Rafae A., Kantamneni L., Sheikh T., Khan R. (2022). Copper deficiency, a rare but correctable cause of pancytopenia: A review of literature. Expert Rev. Hematol..

[36] Kumar N. (2006). Copper Deficiency Myelopathy (Human Swayback). Mayo Clin. Proc..

[37] Kambe T., Tsuji T., Hashimoto A., Itsumura N. (2015). The physiological, biochemical, and molecular roles of zinc transporters in zinc homeostasis and metabolism. Physiol. Rev..

[38] Mousavi S.M., Djafarian K., Mojtahed A., Varkaneh H.K., Shab-Bidar S. (2018). The effect of zinc supplementation on plasma C-reactive protein concentrations: A systematic review and meta-analysis of randomized controlled trials. Eur. J. Pharmacol..

[39] Li B., Cui W., Tan Y., Luo P., Chen Q., Zhang C., Qu W., Miao L., Cai L. (2014). Zinc is essential for the transcription function of Nrf2 in human renal tubule cells in vitro and mouse kidney in vivo under the diabetic condition. J. Cell. Mol. Med..

[40] Oh H.M., Yoon J.S. (2008). Glycemic control of type 2 diabetic patients after short-term zinc supplementation. Nutr. Res. Pract..

[41] Ozawa M., Ninomiya T., Ohara T., Doi Y., Uchida K., Shirota T., Yonemoto K., Kitazono T., Kiyohara Y. (2013). Dietary patterns and risk of dementia in an elderly Japanese population: The Hisayama Study. Am. J. Clin. Nutr..

[42] Canevelli M., Lucchini F., Quarata F., Bruno G., Cesari M. (2016). Nutrition and Dementia: Evidence for Preventive Approaches?. Nutrients.

[43] Rembach A., Doecke J.D., Roberts B.R., Watt A.D., Faux N.G., Volitakis I., Pertile K.K., Rumble R.L., Trounson B.O., Fowler C.J. (2013). Longitudinal analysis of serum copper and ceruloplasmin in Alzheimer’s disease. J. Alzheimer’s Dis..

[44] Bagheri S., Squitti R., Haertlé T., Siotto M., Saboury A.A. (2018). Role of Copper in the Onset of Alzheimer’s Disease Compared to Other Metals. Front. Aging Neurosci..

[45] Wei J., Gianattasio K.Z., Bennett E.E., Stewart J.D., Xu X., Park E.S., Smith R.L., Ying Q., Whitsel E.A., Power M.C. (2022). The Associations of Dietary Copper with Cognitive Outcomes. Am. J. Epidemiol..

[46] Barnard N.D., Bush A.I., Ceccarelli A., Cooper J., de Jager C.A., Erickson K.I., Fraser G., Kesler S., Levin S.M., Lucey B. (2014). Dietary and lifestyle guidelines for the prevention of Alzheimer’s disease. Neurobiol. Aging.

[47] Kobayashi H., Abe M., Okada K., Tei R., Maruyama N., Kikuchi F., Higuchi T., Soma M. (2015). Oral zinc supplementation reduces the erythropoietin responsiveness index in patients on hemodialysis. Nutrients.

[48] Shiota J. (2015). Effect of zinc supplementation on bone formation in hemodialysis patients with normal or low turnover bone. Ren. Fail..

[49] Mokas S., Larivière R., Lamalice L., Gobeil S., Cornfield D.N., Agharazii M., Richard D.E. (2016). Hypoxia-inducible factor-1 plays a role in phosphate-induced vascular smooth muscle cell calcification. Kidney Int..

[50] Voelkl J., Tuffaha R., Luong T.T., Zickler D., Masyout J., Feger M., Verheyen N., Blaschke F., Kuro-o M., Tomaschitz A. (2018). Zinc inhibits phosphate-induced vascular calcification through TNFAIP3-mediated suppression of NF-κB. J. Am. Soc. Nephrol..

